# Angiogenic Modification of Microfibrous Polycaprolactone by pCMV-VEGF165 Plasmid Promotes Local Vascular Growth after Implantation in Rats

**DOI:** 10.3390/ijms24021399

**Published:** 2023-01-11

**Authors:** Ilya Klabukov, Maksim Balyasin, Olga Krasilnikova, Timur Tenchurin, Alexander Titov, Mikhail Krasheninnikov, Daniil Mudryak, Yana Sulina, Alexey Shepelev, Sergei Chvalun, Tatiana Dyuzheva, Anna Yakimova, Dmitry Sosin, Alexey Lyundup, Denis Baranovskii, Peter Shegay, Andrey Kaprin

**Affiliations:** 1 Department of Regenerative Medicine, National Medical Research Radiological Center, 249031 Obninsk, Russia; 2 Obninsk Institute for Nuclear Power Engineering, National Research Nuclear University MEPhI, 115409 Obninsk, Russia; 3 Department of Urology and Operative Nephrology, Peoples’ Friendship University of Russia (RUDN University), 117198 Moscow, Russia; 4 National Research Centre “Kurchatov Institute”, 1, Akademika Kurchatova pl., 123182 Moscow, Russia; 5 City Clinical Hospital No. 67 of Moscow Health Department, 2/44, Salyama Adilya St., 123423 Moscow, Russia; 6 Lomonosov Institute of Fine Chemical Technologies, Russian Technological University MIREA, 119454 Moscow, Russia; 7 Department of Hospital Surgery, Sklifosovsky Institute of Clinical Medicine, Sechenov University, 119435 Moscow, Russia; 8 Department of Obstetrics and Gynecology, Sechenov University, 119435 Moscow, Russia; 9 A. Tsyb Medical Research Radiological Center—Branch of the National Medical Research Radiological Center, Koroleva St. 4, 249036 Obninsk, Russia; 10 Center for Strategic Planning and Management of Biomedical Health Risks of the Federal Medical Biological Agency, 125371 Moscow, Russia

**Keywords:** angiogenesis, material functionalization, electrospinning, gene-activated scaffold, Neovasculgen, physiological relevance, polycaprolactone, regenerative medicine, tissue engineering, vascularization

## Abstract

Insufficient vascular growth in the area of artificial-material implantation contributes to ischemia, fibrosis, the development of bacterial infections, and tissue necrosis around the graft. The purpose of this study was to evaluate angiogenesis after implantation of polycaprolactone microfiber scaffolds modified by a pCMV-VEGF165-plasmid in rats. Influence of vascularization on scaffold degradation was also examined. We investigated flat microfibrous scaffolds obtained by electrospinning polycaprolactone with incorporation of the pCMV-VEGF-165 plasmid into the microfibers at concentrations of 0.005 ng of plasmid per 1 mg of polycaprolactone (0.005 ng/mg) (LCGroup) and 0.05 ng/mg (HCGroup). The samples were subcutaneously implanted in the interscapular area of rats. On days 7, 16, 33, 46, and 64, the scaffolds were removed, and a histological study with a morphometric evaluation of the density and diameter of the vessels and microfiber diameter was performed. The number of vessels was increased in all groups, as well as the resorption of the scaffold. On day 33, the vascular density in the HCGroup was 42% higher compared to the control group (*p* = 0.0344). The dose-dependent effect of the pCMV-VEGF165-plasmid was confirmed by enhanced angiogenesis in the HCGroup compared to the LCGroup on day 33 (*p*-value = 0.0259). We did not find a statistically significant correlation between scaffold degradation rate and vessel growth (the Pearson correlation coefficient was ρ = 0.20, *p*-value = 0.6134). Functionalization of polycaprolactone by incorporation of the pCMV-VEGF165 plasmid provided improved vascularization within 33 days after implantation, however, vessel growth did not seem to correlate with scaffold degradation rate.

## 1. Introduction

Insufficient vascular growth in the area of artificial-material implantation may cause ischemia, fibrosis, the development of bacterial infections, and tissue necrosis around the graft [1,2,3]. The implant area should be activated by angiogenic growth factors to avoid vascular complications and stimulate new tissue growth [4,5]. Targeted delivery and release of pro-angiogenic molecules is one of the key challenges in precision medicine [6,7,8]. One of the solutions to improve the rate of vascularization is biological modification of a graft with biomolecules and cells [9,10], which may lead to a reduction in inflammation and ischemia in affected tissues [11,12].

The timing of stimulation of vascular growth is crucial for successful outcomes after graft implantation. Stimulation of angiogenesis has been shown to be especially important in the early period after scaffold implantation [13,14,15,16]. Previously, it was shown that the use of protein growth factors (fibroblast growth factors, epidermal growth factor, vascular endothelial growth factor (VEGF), platelet-derived growth factor, and other pro-angiogenic biomolecules) and gene therapy drugs, when applied locally, deposited on the surface of the material or embedded in the volume, promoted vascular growth in an implant [17,18,19]. The advantages of prolonged drug release from the polymer volume were observed when the drug was absorbed in the material and chemically attached to a surface or locally administered into a scaffold volume [9,20,21].

Modification of materials with proteins may lead to the appearance of unconventional specificity and immunogenicity [22,23]. On the contrary, the use of drugs based on non-immunogenic molecules (for example, nucleic acids) is a promising direction for implant functionalization [23]. For example, the VEGF165 plasmid with cytomegalovirus (CMV) promoter as a gene therapeutic drug (pCMV-VEGF165) is used to stimulate therapeutic angiogenesis in lower-limb ischemia [24,25]. Techniques for functionalization of material surfaces with plasmid-based gene therapy were used in bone implants [26,27,28].

Previously, an angiogenic effect represented by an increase in vascular density was observed after implantation of microfibrous PCL sheets functionalized with the pCMV-VEGF165 plasmid [18,29]. The important thing is that the enhanced vascular growth by pHGF/VEGF plasmid injection is achieved after a single injection of ‘naked’ DNA without any technique to facilitate DNA uptake such as low-voltage pulses, ultrasound, or liposomes [30]. The short-term effects of angiogenic stimulation in engineered tissues were noted and may not be sufficient to ensure good vascular organization in the long term, as the vasculature is likely to revert to a disorganized state upon implantation without additional cues [31]. Therefore, it is critically important to continue developing in vitro and in vivo methods to evaluate degree and pattern of vascularization.

Hydrogels, polycaprolactone, polylactic acid, and other polymers are used as drug carriers to achieve prolonged drug release [17,32]. The implantation of biodegradable materials with biomolecules for various purposes leads to the release of molecules during the material resorption [33,34]. The choice of material for modification is important due to the differences in biocompatibility and biodegradation, which affect the timing and rate of drug release from the material [35].

Biodegradable materials have several advantages associated with the elimination of artificial material from the implantation zone and its replacement with the native tissue of the recipient [36,37]. However, in the early postoperative period, the application of biodegradable materials can lead to functional graft failure and other adverse events in the case of intensive biodegradation [38,39]. The side effects and complications of graft implantation may be caused by undiscovered properties of materials and interaction of the scaffold with surrounding tissues. The physiological relevance of biocompatible scaffolds is usually achieved by formation of smart scaffolds with prolonged or controlled release of biomolecules [40]. At the same time, interaction of tissues with biocompatible metabolites may also have a delayed effect on the physiological response in a tissue-specific and cell-specific manner.

Interestingly, an excess of monomers may change the mechanical properties and may auto-catalyze the hydrolysis resulting in altered degradation kinetics [41]. Han and Pan (2009) created a model of polymer degradation showing the interplay between the autocatalytic hydrolysis reaction, oligomer diffusion, and degradation-induced crystallization [42,43]. However, stages and rates of polyester degradation after implantation remain uncovered. The same autocatalytic effect of hydrolysis degradation has essential meaning at early stages of degradation of polymers [44]. The initial stage of destruction is a bulk degradation phase during which enzyme-enriched physiological fluids penetrate the entire polymer bulk, causing hydrolysis. An acidic gradient may be produced due to the internal concentration of auto-catalysis product [45], moreover, an acidic gradient may lead to diffusion of impregnated biomolecules from the volume of the polymer. It is known that degradation of the polymer occurs faster under in vivo conditions compared to simulated conditions in vitro, however, enzymatic activity outside of the digestive system is not sufficiently studied [41]. Moreover, copolymers may also facilitate host-dependent and tissue-specific degradation depending on enzymatic activity and concentrations. For example, phosphate ester bonds may be catalytically degraded by alkaline phosphatase, which is highly expressed in bone tissue [46].

One of the important but insufficiently studied issues is the effect of the growth rate of new vessels on the rate of scaffold degradation. While there are many studies on the angiogenic modification of biomaterials, as well as studies on material resorption, works assessing dependence of the material resorption on the rate of vascularization are rare.

The purpose of this study was to evaluate the angiogenic effect of a microfiber polycaprolactone scaffold modified with different concentrations of the pCMV-VEGF165 plasmid after implantation in rats and to study the effect of the number of newly formed vessels on the rate of scaffold degradation.

## 2. Results

### 2.1. Morphology and Biocompatibility

**Figure 1:**
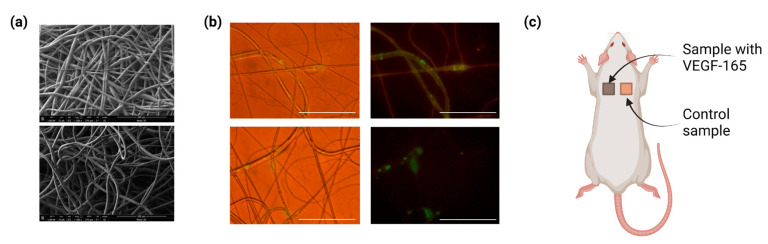
(**a**) Microfibers with incorporated biomolecule-containing buffer, SEM and (**b**) emulsion electrospray-derived microfibers with GFP incorporated: light and fluorescence microscopy. Figure adapted from Tenchurin et al. (2017) with permission of the publisher [33]. (**c**) Surgery scheme for implantation of microfibrous PCL-based scaffolds. Created with BioRender.com.

Obtained polycaprolactone scaffolds had a microfibrous pattern (Figure 1a) and contained bubbles with the pCMV-VEGF165 plasmid included in the volume of fibers, as previously visualized by fluorescence microscopy with the use of green fluorescent protein (GFP) incorporation (Figure 1b). Morphological assessment of fibrous samples was performed by scanning electron microscopy (SEM), and shown in Table 1.

The morphological study of the explanted scaffolds (Figure 1c) with surrounding tissues in all groups showed no signs of infiltration by leukocytes on days 7, 16, 33, 46, and 64. The presence of foreign-body giant cells was observed in the tissues. The absence of signs of an acute inflammatory process and rejection of the material indicates minimal cytotoxicity of the material under in vivo conditions (see Data Availability Statement).

### 2.2. Angiogenesis

Explanted samples (tissue flap with partially degenerated scaffold) were examined on days 7, 16, 33, 46, and 64. On day 7, the autopsy material was described only qualitatively (without counting the vessels) due to the still underdeveloped tissue around the preserved fibers of the implanted scaffold. Approximately 80% of the scaffolds were not fragmented; the vessels were mostly on the periphery of the preserved matrix.

On day 16, the density of the vessels was higher in the HCGroup and the LCGroup compared to the control group (by 8%, no significance), which may be explained by the release of the VEGF165 plasmid from the polymer volume.

**Figure 2:**
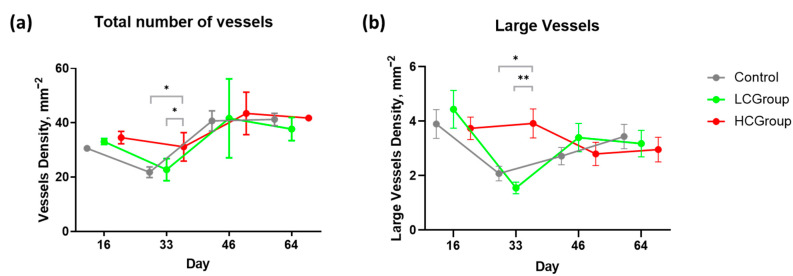
(**a**) Differences in the total number of blood vessels per field of view in groups and (**b**) differences in the number of large blood vessels per field of view in groups. Data presented as mean ± 95% CI. LCGroup—low-concentration group, HCGroup—high-concentration group. Note: * *p* < 0.05, ** *p* < 0.01.

On day 33, vessel density in the HCGroup was 42% higher compared to the control group (*p*-value = 0.0344) and 45% higher compared to the LCGroup (*p* = 0.0259), which may be explained by the further release of the VEGF165 plasmid from the volume of polycaprolactone microfibers during the process of resorption. However, on day 33, there was a decrease in vessel density in all groups compared to day 16. After day 33, there was a trend towards increase of vessel density, but on days 46 and 64 the difference in the number of blood vessels between all groups did not reach statistical significance (Figure 2a, Tables S1 and S2).

The percentage of large vessels was statistically significantly higher in the HCGroup on day 33 compared to the control group (*p* = 0.0457) and the LCGroup (*p* = 0.0043) (Figure 2b, Tables S3 and S4). On days 16, 46, and 64 the difference in the number of large blood vessels between all groups did not reach statistical significance.

### 2.3. Biodegradation

**Figure 3:**
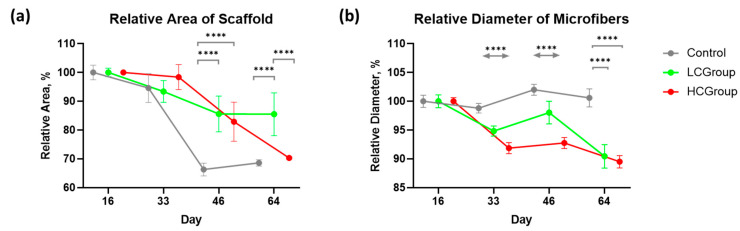
Changes in the implanted scaffolds in the control group and in groups modified with different concentrations of the VEGF165 plasmid: (**a**) Relative area of implanted scaffolds and (**b**) relative diameter of fibers in implanted scaffolds. Data presented as mean ± 95% CI. Note: **** *p* < 0.0001.

There was a gradual decrease in visible material as a result of fragmentation and resorption. Quantitative assessment revealed that a decrease in the area and average diameter of polymer microfibers (except for Control group) was observed in the explanted materials due to the material bioresorption (Figure 3).

Analysis of the decrease of relative area of microfibrous samples showed that the resorption of the control samples was more intense compared to the LCGroup and the HCGroup on day 46 and compared to LCGroup on day 64 (*p*-value < 0.0001) (Figure 3a, Table S5). However, in the HCGroup, there was a significant decrease in fiber diameter compared to the LCGroup (*p*-value < 0.0001) and control group (*p*-value < 0.0001) (Figure 3b, Table S6). At the same time, in the control group, opposite results were observed when estimating the area of scaffolds and when estimating the fiber diameter. In the control group, the area of the scaffold was significantly less than in the LCGroup (Figure 3a), but the fiber’s diameter was larger than in the LCGroup and HCGroup on day 64 (Figure 3b). This may be partially explained by the fact that the fibers of the scaffolds in the control group did not contain bubbles in contrast to scaffolds modified by VEGF165 plasmid. Bubbles are known to reduce the volume of the fiber and to make it more prone to biodegradation. In contrast, the assessment of the surface area of the scaffold showed significant degradation of control samples.

We examined the correlation between the number of vessels and the rate of material bioresorption and found no statistically significant correlation between the number of vessels and the rate of material bioresorption (the Pearson correlation coefficient was ρ = 0.20, *p*-value = 0.6134).

## 3. Discussion

Previously, implantation of scaffolds vitalized by plasmids encoding vascularization growth factors was shown to have the same physiological effect as the use of recombinant protein growth factors [18,34,47]. The approach to the implantation of gene-activated materials opens the new age of cell-free regenerative medicine [29].

The timing of graft vascularization is crucial for the successful transplantation of organs, tissues, and tissue-engineered grafts [15]. In the present work, we studied the dynamics of vessel growth at different time points. The application of scaffolds enriched with VEGF165-plasmid resulted in the formation of more vessels compared to the control group on day 16, but this result was not statistically significant. However, on day 33, there was a statistically significant increase in vessel density in the group with a high concentration of VEGF165-plasmid in the scaffold’s fibers compared to the control group and the group with a low concentration of VEGF165-plasmid. On days 46 and 64, there were no differences in vessel density between the groups. Our results showed that scaffolds with high concentration of VEGF165-plasmid were capable of accelerating novel vessel growth in comparison with low-concentration scaffolds and control samples. These results suggest that the technique of enriching fibrous scaffolds with plasmids has the potential for stimulation of vascularization, but further studies are needed to improve this approach.

In our study, we used plasmids incorporated into the volume of the scaffold. Thus, the time of actual protein expression was delayed due to the period needed for scaffold degradation, transfection, intracellular transport, transcription, and translation [48]. Expression of protein after injection of plasmid DNA typically lasts 10–14 days in most mammalian tissues [49].

Considering the length (4559 bp) and molar mass (~2.93 MDa) of the pCMV-VEGF165 plasmid and the molar mass of the VEGF165 protein (19 kDa), if we take the ratio of plasmid to secreted protein as 1:1, the concentration of VEGF165 protein will increase in the sample volume by 0.063 × (19 × 10^3^/2.93 × 10^6^)/(0.05 mL) = 8.2 pg/mL for the LCGroup, and by 95.6 pg/mL for the HCGroup. However, injection of the naked plasmid DNA provides a long-term expression of secreted proteins [50,51], which makes precise calculations impossible.

In humans, the increase of VEGF165 concentration from 26 pg/mL to 312 pg/mL led to an increase in vessel density of 229% [52]. In rats, the increase of VEGF concentration in tissues samples from 145 pg/g (control samples) to 520 pg/g led to an increase in vascular density of 16% [53], which allows us to estimate the level of VEGF in rat’s intact skin tissues as 100 pg/mL. This value is compatible with the model calculated concentrations (8.2 pg/mL for samples from the LCGroup and 95.6 pg/mL for samples from the HCGroup), therefore, physiological effects could be expected.

**Figure 4:**
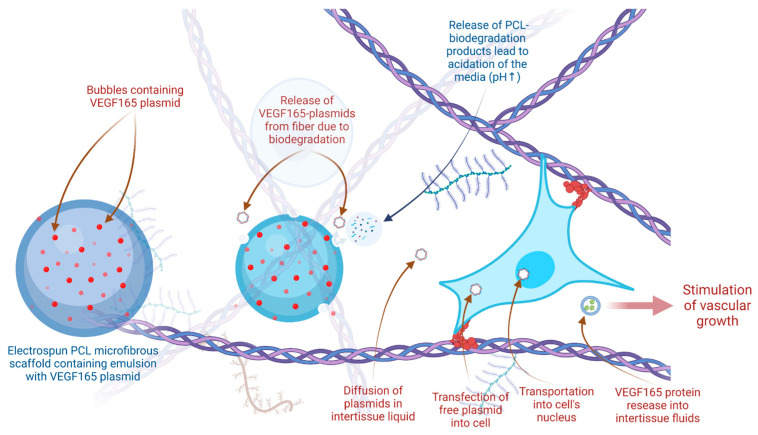
Mechanisms of non-viral delivery for angiogenic stimulation.

The results obtained in our study are consistent with those in other works on angiogenic modification of scaffolds. For example, vessel density was 22 ± 6 mm^−2^ in implanted control collagen matrix compared with 30 ± 5 mm^−2^ in collagen matrix vitalized with bFGF (increase of 35% in the HCGroup comparison with the LCGroup and CGroup) [54]. The use of pre-vascularized alginate grafts showed an increase in vascular density from 25 to 60 mm^−2^ on day 7 after implantation. At the same time, vascularization peaked on days 10–14, which was confirmed by the investigated stages of angiogenesis [55,56,57]. Our study showed that vascular growth may also be promoted by the release of VEGF165 plasmid from the scaffold (Figure 4). We also demonstrated a dose-dependent effect on day 33 when vessel density was significantly higher in the group with a high VEGF165 plasmid concentration compared to the low-concentration group and the control group.

One of the notable findings of our study was the decrease in the total number of vessels on day 33 compared to day 16 (Figure 2a). This decrease was noted in all groups. We speculate that reduction in the total number of vessels may be related to the phenomenon of blood vessel regression noted in some studies during remodeling of vascular plexus [58]. However, the fact of vessel number reduction shown in our study, needs further investigation to clarify reasons for such dynamics. Initially, we supposed, on this basis, that the regression of total number of vessels could be attributed to the regression of small vessels associated with the maturation of large vessels. However, instead of a gradual increase in the number of large vessels, a decrease in the number of large vessels on day 33 compared to day 16 was also observed in the control group and in the group with low plasmid concentration. In the group with high plasmid concentration, the number of large vessels did not change or even slightly increased and was significantly higher compared to other groups. Our results showed curious changes in the total number of vessels and in the number of large vessels (with a diameter greater than 15 μm), warranting further investigation of the dynamics of vascular network formation during the implantation of various scaffolds.

We showed that there were statistically significant differences in scaffold degradation between experimental groups and the control group. However, we showed no statistically significant correlation between the number of vessels and the rate of material bioresorption (the Pearson correlation coefficient was ρ = 0.20, *p*-value = 0.6134). We hypothesized that the effect of the number of vessels on the scaffold degradation may be delayed.

Our results showed that scaffolds with high concentration of plasmid had significantly decreased fiber diameter compared to the LCGroup and Control group on days 33 and 46, and compared to Control group on day 64. This may possibly be explained by the fact that the fibers of the scaffolds in the HCGroup contained bubbles in contrast to control scaffolds. Bubbles are known to reduce the volume of the fiber and make it more prone to biodegradation. However, this question requires further investigation.

The possible ways to explain the contradicting results of degradation of control samples may include properties of control samples (fibers without modification), or the specific physiological response to microfibrous scaffolds. The features of physiological response may be caused by maturation of tissues around the fibers with different properties. Furthermore, it is necessary to take into account that, if an implant causes a substantial immune reaction, the thickness of the surrounding connective tissue capsule increases. In this case, the blood supply to the capsule remains insufficient, leading to faster degradation of scaffold. Poor blood supply and lymphatic insufficiency in the capsule lead to the temporary accumulation of biodegradation products in the capsule, which was previously observed in various studies [59,60,61,62].

The phenomenon of autocatalytic degradation consists in the acceleration of the degradation of polymers when exposed to the hydrolysis products of oligomers that remain inside the material, causing a local decrease in pH, which accelerates the degradation rate (Figure 4a). Thus, the ε-hydroxycaproic acid (monomer formed during the biodegradation of PCL) is included in the tricarboxylic acid cycle and is oxidized to the final products of CO_2_ and water, and completely eliminated from the body, and during degradation in a closed volume, the autocatalytic degradation mechanism begins to take a greater role in the polymer decomposition compared to hydrolytic degradation. The previously observed effect of inhibition of angiogenesis after implantation of porous PCL can be caused primarily by the understudied effect of inclusion of biodegradation metabolites in the Krebs cycle of the cells in surrounding tissues that can lead to inflammatory response [63].

Due to the autocatalytic action of carboxylic acids, chains terminated by hydroxyl groups are much more stable than those terminating with carboxylic acids [64]. Catalytic-enhanced degradation led to carboxylic end-groups autocatalysis, the process with mass loss beginning from massive cleavage of the polymer backbone covalent bonds, resulting in a loss of material integrity [41,65].

**Figure 5:**
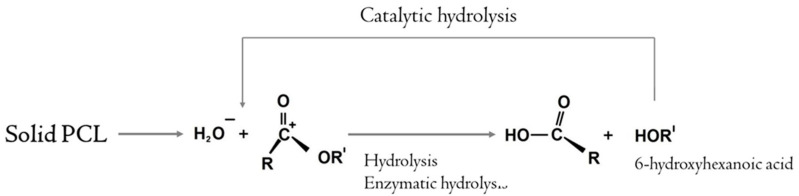
Scheme of ester bond hydrolysis in PCL-microfibers and free-radical-enhanced autocatalysis.

Based on potential mechanisms of PCL-radical-mediated addition and cleavage [41], we present a scheme of hydrolysis-mediated autocatalytic degradation (Figure 5).

In vivo resorption of polymers occurs faster than in vitro due to the effects of body metabolites, as well as due to the immunological responses to the implant [66,67,68]. The main mechanisms of biodegradation of samples from polymeric materials in the body are the hydrolysis of the ester groups contained in them and the oxidative degradation by active oxygen radicals [69]. In the process of hydrolysis, the polymer chains of polyesters are destroyed, as a result of which the mechanical properties of the frameworks decrease and the mass loss gradually increases. The presence of microorganisms or eukaryotic cells significantly affects the rate of change in the molecular weight of polyesters [70,71]. The autocatalytic nature of degradation is caused by the local accumulation of acids released during polyester breakdown and is observed both in vitro and in vivo [72,73]. Thus, in aqueous media, the ester bonds of the polymeric material break throughout the fiber volume, causing the molecular weight and mechanical properties of the samples to decrease. The scaffold micro-architectonics may also affect the response on biodegradable materials. Indeed, faster degradation of shifted constructs may be attributed to their tortuosity, making them less permeable and prone to degradation as the result of the accumulation of acidic products in the tortuous architecture of the samples [74]. The destruction of polymer fibers to a multicomponent mixture of biodegradation products is not a fully-studied field, and the use of modern biochemistry tools is required to study the composition of extracellular fluid in tissues. The results of the in vivo evaluation of the PCL-scaffold bioresorption in vivo showed that the scaffold was resorbed by 8–12% within 64 days after implantation, which may be explained not only by the enhancement of endogenous enzymatic activity in the rat organism [75], but also by the possible compaction of connective tissue in the zone of contact with the material.

Our in vivo study of the resorption dynamics showed that the microfiber bioresorption rate was higher in scaffolds with a high concentration of the VEGF165 plasmid compared to scaffolds with a low plasmid concentration on day 46, which may possibly be explained by the delayed effects of intensive vascular growth in the implantation zone in the HCGroup noted on day 33. The results of degradation of the control samples showed the need for the detailed investigation of physiological responses to polymeric materials.

The limitations of this study are the small amount of samples and the method of quantitative morphological evaluation. Differences in the number of vessels between the LCGroup and control group appeared to be statistically insignificant. This may indicate both a low effect of low dose and insufficiency of the morphological evaluation.

## 4. Materials and Methods

### 4.1. VEGF165-Modified Implants

The electrospinning machine was used to derive microfibrous samples (Figure 1a). We prepared microfiber scaffolds (1 cm × 1 cm in size, 500 ± 15 μm thick and 2–3 μm fiber diameter) by electroforming of polycaprolactone (PCL) containing a water emulsion of the VEGF165 plasmid (Figure 1b,c). Within the emulsion electrospinning process, the pCMV-VEGF165 plasmid (gene therapy drug Neovasculgen, NextGen LLC) was incorporated into fibrous PCL materials at two concentrations: low concentration (LCGroup)—0.005 mg/mL in buffer, or 0.005 ng of VEGF165 plasmid per 1 mg of fibrous PCL, and high concentration (HCGroup)—0.05 mg/mL in buffer, or 0.05 ng of VEGF165 plasmid per 1 mg of fibrous PCL. Microfiber scaffolds without modifications were used as the control group (CGroup). Morphological characteristics of scaffolds were evaluated based on images derived by scanning electron microscopy. Previously, we obtained microfibrous PCL-grafts containing epidermal growth factor (EGF) proteins in the volume of fibers using this technique [33].

### 4.2. Animals

We implanted microfibrous scaffolds subcutaneously in the interscapular area of female Wistar rats (*n* = 24) with an initial body weight of 180–200 g at 2 months of age (Figure 1d). Anesthesia was performed with Zoletil-100 (Virbac, Carros, France) and Rometar (Bioveta, Ivanovice na Hané, Czech Republic). On days 7, 16, 33, 46, and 64, the animals were sacrificed by decapitation under anesthesia. The scaffolds were explanted with surrounding tissue and assessed, as described below for the subsequent morphological study.

### 4.3. Histology

For histological studies, the samples were fixed in 10% buffered neutral formalin, washed with running water, dehydrated, and embedded in paraffin. Sections 4 μm thick were obtained with a Microm HM 355 s microtome (Thermo Fisher Scientific, Waltham, MA, USA), stained with hematoxylin and eosin, and microscopy was performed with the Pannoramic DESK scanning system (3DHistech Ltd., Budapest, Hungary).

### 4.4. Biocompatibility

The biocompatibility of the material was assessed based on the observation of the histology of the H&E-stained specimens: the number of immune cells in the area of material implantation was qualitatively assessed, and the toxicity was determined by the number of neutrophils.

### 4.5. Angiogenesis

The density of the vessels and the percentage of large vessels in the area of resorption of the implanted material were counted manually using the blind method together with the staff of the experimental morphology laboratory of the Sechenov University; five investigators in total were involved. The density of vessels was determined as the number of vessels per 1 mm^2^ of observation area on microphotography. Vessels with a diameter greater than 15 μm were considered as large vessels. Data on the number and size of vessels were counted in five fields of view with a diameter of 600 μm each in the program Pannoramic Viewer v.1.15.4.).

### 4.6. Quantitative Assessment of the Bioresorption Dynamics of the Material

The evaluation of the dependence between the degree of vitalization of the scaffold with VEGF165 plasmid and the change in the mean diameter of microfibers after implantation in rats was carried out by automatic histological analysis of H&E-stained sections using QuPath v0.4.0 software.

### 4.7. Statistics

Statistical analysis was performed in the R v4.1.0. Data on the number of blood vessels (overall and large vessels) per slide were collected independently by two morphologists, data collected in at least 7 fields (up to 15 depending on the size of the slice) with an area of 0.2827 mm^2^ each in 2 to 5 biological replicates. To compare the calculated number of vessels per area between groups over time, Poisson GLMM models were built, where the fixed factors were time and group, and the random factor was the animal. The model was tested for overdispersion. Data are presented without adjustment for multiple comparisons. The effect of the type of scaffold modification on the biodegradation of the material was determined by two-way ANOVA. Differences were considered significant at *p*-value < 0.05.

### 4.8. Ethics

All manipulations with laboratory animals were approved by the Local Ethics Committee of the Sechenov University and were carried out in accordance with the bioethic rules approved by the European Convention for the Protection of Vertebrate Animals Used for Experimental and Other Scientific Purposes.

## 5. Conclusions

We believe that the vitalization of a scaffold with DNA plasmid is a promising alternative to the enrichment of scaffolds with growth factors. Our point of view is supported by the following results of the present study:

(1) Microfibrous polycaprolactone scaffolds can be obtained by emulsion electrospinning and enriched with the gene-therapeutic drug Neovasculgen containing the pCMV-VEGF165 plasmid.
(2) After subcutaneous implantation in rats, an increase in the vascular density was observed in microfibrous scaffolds enriched by the plasmid. Scaffolds with high concentration of pCMV-VEGF165 plasmid (0.05 ng of plasmid per 1 mg of PCL) accelerated vascularization compared to scaffolds with low plasmid concentration and control scaffolds. The vascular density on day 33 was 42% higher compared to the Control group (*p*-value = 0.0344).
(3) The dose-dependent effect was shown between the concentration of the pCMV-VEGF165 plasmid and the vascularization on day 33. The effect was reversible and on day 46 the values in experimental groups did not differ from the control group. The dynamics of vascularization show that gene-activated materials may participate in delayed and prolonged physiological responses.
(4) Microfiber bioresorption rate was higher in scaffolds with a High concentration of the VEGF165 plasmid compared to scaffolds with a Low plasmid concentration on days 33 and 46. However, we have not found a statistically significant correlation between scaffold degradation rate and vessel growth.

Our results demonstrated the efficacy of angiogenic modification of synthetic scaffolds to improve the physiological relevance of tissue-engineered grafts. The post-implantation interactions of growing tissues and implanted materials need further investigation.

## Figures and Tables

**Table 1:** Characteristics of fibrous scaffold samples.

Day	Type of Sample	Concentration of Neovasculgen (VEGF165 Plasmid) in Buffer (Ratio of the Mass of VEGF165 Plasmid per Mass of PCL Scaffold)	Porosity, %	Mean Pore Size, μm	Mass of Sample (Mean ± SD), mg	Mass of VEGF165 Plasmid per Sample, ng
1	Control	No	91.1	54	7.9 ± 2.2	0
2	Low concentration	0.005 μg/mL (0.005 ng/mg)	90.8	46.5	12.5 ± 2.9	0.063 ± 0.015
3	High concentration	0.05 μg/mL (0.05 ng/mg)	93.5	36	14.7 ± 3.7	0.74 ± 0.19

## Data Availability

The data presented in this study are openly available in FigShare at https://doi.org/10.6084/m9.figshare.21779348.

## Supplementary Materials

The following supporting information can be downloaded at: https://www.mdpi.com/article/10.3390/ijms24021399/s1.
