# Supplementary material for: Angiogenic Modification of Microfibrous Polycaprolactone by pCMV-VEGF165 Plasmid Promotes Local Vascular Growth after Implantation in Rats
Source: Int J Mol Sci. 2023 Jan 11;24(2):1399. doi: 10.3390/ijms24021399 (PMC9865169; doi:10.3390/ijms24021399)
Supplement: Supplementary file 1 [file ijms-24-01399-s001.zip › ijms-2065270-supplementary.pdf]

Supplementary Materials

Table S1. Vessel density per 1 mm^2.

Day	Control Group (CGroup), mm^{-2}	Low Concentration Group (LCGroup), mm^{-2}	High Concentration Group (HCGroup), mm^{-2}
16	8.61 (6.79–10.93)	9.31 (6.99–12.41)	9.33 (7.78–11.19)
33	6.23 (5.00–7.77)	6.11 (4.90–7.62)	8.83 (6.97–11.19)
46	10.99 (9.18–13.16)	11.30 (8.76–14.56)	11.77 (9.14–15.16)
64	12.04 (9.79–14.79)	8.74 (6.73–11.33)	11.10 (8.61–14.31)

Data represented as Poisson μ ± 95%CI

Table S2. Vessel density: Group comparisons.

Day	LCGroup vs. CGroup	HCGroup vs. CGroup	HCGroup vs. LCGroup
16	1.081 (0.745–1.57, p = 0.6831)	1.083 (0.803–1.46, p = 0.6010)	1.002 (0.714–1.41, p = 0.9900)
33	0.981 (0.718–1.34, p = 0.9043)	1.417 (1.026–1.96, p = **0.0344**)	1.445 (1.045–2.00, p = **0.0259**)
46	1.028 (0.753–1.40, p = 0.8644)	1.071 (0.785–1.46, p = 0.6646)	1.042 (0.728–1.49, p = 0.8203)
64	0.726 (0.521–1.01, p = 0.0585)	0.923 (0.665–1.28, p = 0.6289)	1.271 (0.883–1.83, p = 0.1964)

Data represented as Poisson ratio ± 95%CI, significant p-values marked bold

Table S3. Large blood vessel count.

Day	Control Group	Low Concentration Group	High Concentration Group
16	3.90 (2.489–6.11)	4.44 (2.583–7.64)	3.74 (2.633–5.31)
33	2.08 (1.359–3.19)	1.55 (0.985–2.43)	3.92 (2.500–6.15)
46	2.72 (1.866–3.96)	3.40 (2.020–5.73)	2.80 (1.654–4.75)
64	3.44 (2.247–5.26)	3.18 (1.881–5.37)	2.96 (1.750–5.00)

Data represented as Poisson μ ± 95%CI

Table S4. Large blood vessel count: Group comparisons.

Day	LCGroup vs. CGroup	HCGroup vs. CGroup	HCGroup vs. LCGroup
16	1.138 (0.563–2.30, p = 0.7182)	0.958 (0.542–1.69, p = 0.8837)	0.842 (0.442–1.61, p = 0.6012)
33	0.743 (0.399–1.38, p = 0.3477)	1.881 (1.012–3.50, p = **0.0457**)	2.533 (1.339–4.79, p = **0.0043**)
46	1.251 (0.658–2.38, p = 0.4942)	1.031 (0.540–1.97, p = 0.9253)	0.824 (0.393–1.73, p = 0.6099)
64	0.925 (0.471–1.82, p = 0.8206)	0.861 (0.438–1.69, p = 0.6639)	0.931 (0.443–1.96, p = 0.8499)

Data represented as Poisson ratio ± 95%CI, significant p-values marked bold

Table S5. Area of scaffold.

Day	Control	Low conc. VEGF-165	High conc. VEGF-165
16	28 (25–31)	29 (25–33)	26 (24–29)
33	26 (24–28)	27 (25–30)	26 (23–29)
46	18 (16–20)	25 (22–27)	22 (19–25)
64	19 (17–21)	25 (21–28)	19 (16–21)
Area % with 95% CI			

Table S6. Average diameter of microfibers.

Date	Control	Low conc. VEGF-165	High conc. VEGF-165
16	19.6 (19.4–19.9)	20.1 (19.9–20.3)	20.4 (20.3–20.5)
33	19.6 (19.5–19.8)	19.4 (19.2–19.6)	18.7 (18.4–18.9)
46	20.3 (20.1–20.5)	19.5 (19.3–19.8)	18.6 (18.4–18.8)
64	19.8 (19.4–20.2)	18.1 (17.3–19.0)	17.7 (17.5–18.0)
Mean (μm) with 95% CI			
